# Atomic Force Microscopy Study of Nano-Physiological Response of Ladybird Beetles to Photostimuli

**DOI:** 10.1371/journal.pone.0012834

**Published:** 2010-09-22

**Authors:** Natalia V. Guz, Maxim E. Dokukin, Igor Sokolov

**Affiliations:** 1 Department of Physics, Clarkson University, Potsdam, New York, United States of America; 2 Nanoengineering and Biotechnology Laboratories Center (NABLAB), Clarkson University, Potsdam, New York, United States of America; Dalhousie University, Canada

## Abstract

**Background:**

Insects are of interest not only as the most numerous and diverse group of animals but also as highly efficient bio-machines varying greatly in size. They are the main human competitors for crop, can transmit various diseases, etc. However, little study of insects with modern nanotechnology tools has been done.

**Methodology/Principal Findings:**

Here we applied an atomic force microscopy (AFM) method to study stimulation of ladybird beetles with light. This method allows for measuring of the internal physiological responses of insects by recording surface oscillations in different parts of the insect at sub-nanometer amplitude level and sub-millisecond time. Specifically, we studied the sensitivity of ladybird beetles to light of different wavelengths. We demonstrated previously unknown blindness of ladybird beetles to emerald color (∼500nm) light, while being able to see UV-blue and green light. Furthermore, we showed how one could study the speed of the beetle adaptation to repetitive flashing light and its relaxation back to the initial stage.

**Conclusions:**

The results show the potential of the method in studying insects. We see this research as a part of what might be a new emerging area of “nanophysiology” of insects.

## Introduction

Insects, being the most numerous and diverse group of animals on Earth, are highly efficient bio-machines varying greatly in size. Many species of insects are predators, parasitoids, and pollinators and are important in agriculture. Examples include pollinators such as honeybees and other wild bees, and ladybird beetles as predators. Some are major agricultural pests and are major competitors with humans for crops. Mosquitoes and various other insects are vectors of plant, animal, and human diseases. Vast lands on the planet are underdeveloped being excessively populated by blood-sucking insects. Those are only a few reasons of why the study of insects is quite an active area of research [1]. Recent studies in insect physiology continue to reveal new mechanisms in respiration [2], [3], communication [4] and other aspects of insect behavior and function. At the same time, little exploration has been done with modern nanotechnology tools.

Atomic force microscopy (AFM) is one of the major techniques, which has been instrumental in the emergence of what is called nowadays “nanotechnology”. The AFM technique has become popular in the study of biological materials at the nanoscale [5], [6], [7]. AFM is based on the detection of forces acting between an AFM probe and sample surface. The probe is attached to a very flexible cantilever. Any motion of the cantilever is detected by various methods. The most popular is an optical system of detection. Laser light is reflected from the cantilever, and its motion is detected by a photodiode. The probe is then brought to a contact, engaged with the surface of interest. Scanning over the surface, the AFM system records the deflection of the cantilever with sub-nanometer precision. If the surface is oscillating itself, the oscillations can be recorded when the scanning over the surface is switched off. As was shown, AFM is capable of measuring motion/oscillation of the surface of biological cells at the level of several nanometers [5], [8], [9], [10], [11]. Expansion of this technique to study more complex living objects, like insects, has been restricted by the maximum vertical motion of the AFM probe that can be measured with the existing AFM setups (typically within 50µm). Organ and body movements in a living insect easily exceed this range. Thus, the use of AFM to record the oscillations of insect's surface requires protection of the integrity of the AFM cantilever. A technical solution was suggested [12] to keep an insect motion partially restricted while recording the AFM signal. It was shown that that method allowed for recording information from the internal live processes of the insect at the subnanometer scale. It was shown that the recorded signals had a much broader spectral range (up to several kHz) than the studied before (up to 5Hz). It was substantially richer than just known breathing, heartbeat cycles [13], [14], [15], [16], coelopulses [17], [18], etc. To compare, a recently described rather sensitive optical detection system [19] allows detection of surface oscillations from an area of ∼500µm^2^ with a noise level of 0.5±0.2 nm root-mean-squared (r.m.s.). The used here AFM method allows addressing the area as small as ∼100nm^2^ (0.0001µm^2^) with an example of noise level of (2±0.2)×10^−3^ nm r.m.s. at the range of frequencies of 60–120Hz. Apparently, the signals corresponding to the higher frequencies, above 5Hz and up to tens of KHz have not been detected when using the previous methods due to their limited sensitivity in the high frequency range. The frequencies of the observed in [12] peaks were substantially higher than the frequencies of breathing, gut peristalsis, coelopulses, or heart beats. Most probably, these signals originate at the contraction of muscles of internal organs.

Here we use the AFM method to study precise internal physiological responses of ladybird beetles when exposed to the external stimulus with light. Using a series of periodically flashing light, we learn the speed of the beetle adaptation to the repetitive flashing light and its relaxation. Studying light of different wavelengths, we study the sensitivity of the beetle to different colors.

## Results and Discussion

**Figure 1:**
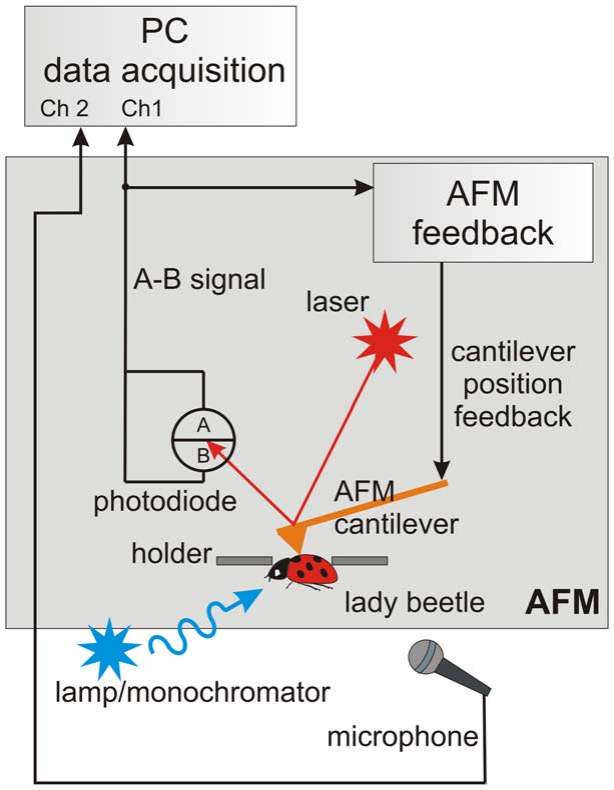
The AFM setup to collect the surface oscillations. An AFM probe is put in contact with an insect surface at a chosen place, and the deflection signal from the AFM cantilever is recorded. Data from a microphone placed near the insect is used for the analysis of external noise. For our stimulus experiments, a flashing monochromatic light was used. The signals are collected on the beetle's elytra in all experiments.

We studied the oscillations of the insect body surface of the ladybird beetle (*Hippodamia convergens*). A schematic of the experiments is shown in Fig. 1. While being roughly positioned with a built-in optical system, an AFM probe can be located on the surface of the insect with nanometer precision. In the stimulus experiments, a flashing monochromatic light was used to illuminate the beetle head. To separate the oscillations coming from the insect from the room noise, and to monitor the constancy of such noise during the course of experiments, a broad band microphone was used to record the room noise. A baseline noise was found as the base line spectra recorded on a dead beetle (this included both the room and instrumental noise).

Let us show how repeating the stimulus, we can study the adaptation of the insect organism to external stimuli, as well as its return to the initial stage. In these experiments, we will analyze the insect adaptation (“learning”) to an external stimulus of flashing light, and its return or relaxation to its initial state (“ forgetting”). Then, we will measure the sensitivity of ladybird beetles to light of different color.

It is important to note the used brightness of light: The intensity of light used in the current experiments was rather low to avoid any fatigue for the beetles. Moreover, excessively bright light could even potentially result in either temporary or permanent blindness of the beetles. The intensity of light was chosen to be as low as possible to reliably see the beetle response (it was certainly lower than a mid-day natural sunlight).

### Adaptation and relaxation experiment with light

**Figure 2:**
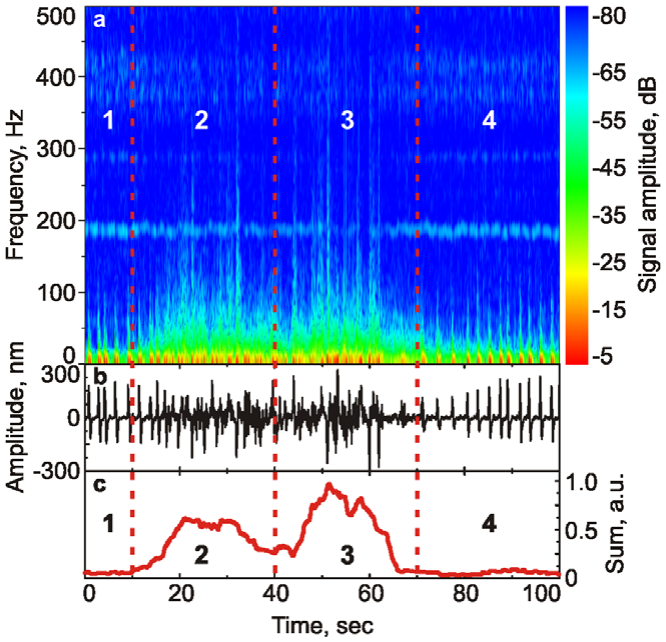
Analysis of the beetle response to a series of flashing light. The flashing light is on in region (2) and off in regions (1,3,4). Altered spectrum relaxes back to the initial level in region 3. (a) Time-dependent spectra (amplitude versus frequency); (b) Row signal as recorded; (c) The average of the Fourier amplitudes with frequencies of the range of 40–100 Hz. The light brightness was adjusted to a sufficiently low-level which presumably just disturbs the insect rather than blinds or fatigues it.

The stimulation was done with flashing (0.5–1 Hz) UV light of 375nm (the beetles are highly sensitive to light of this wavelength [20], see also the below). We found that beetles demonstrate effective changes in the recorded spectra when stimulated with such flashing. It might be the physiological reaction on a possible threat (Changing light can be a warning signal of a possibly approaching predator. Although, we need to say that we did not find the evidence in the literature supporting the hypothesis that flashing light could be treated as a threat by insects except cockroaches). To study the recorded mechanical response of the beetle's surface quantitatively, we analyze their Fourier spectra in time. Fig. 2a shows a typical time series of 0.01–500Hz spectra when a beetle is at rest and when stimulated with the flashing light. One can see the change in the spectrum when the flashing light is on (time is 10 sec) and off (40 sec). The relaxation, which takes place right after switching the light off, shows comparable and sometimes even stronger disturbance of spectra, region 3 of Fig. 2a. Region 4 (70–100 sec in Fig. 2) shows the “relaxed” spectra that is close to the initial one. Fig. 2b shows the amplitude of the recorded signal given the entire 100 seconds. One can see randomized spectrum in regions 2 and 3. Fig. 2c shows the Integral spectra, and the sum of the Fourier amplitudes with frequencies of 40–100 Hz. One can see that this signal may serve as a good indicator of the beetle's reaction to external stimuli.

It is interesting to note that shown in Fig. 2 reaction of the beetles after the flashing series (region 3) was stronger than during the flashing (region 2). This might be explained by a possibly scaring action of the flashing light. It is natural for insects to become still when scared. After the “danger” is gone, they try to escape/move to a safer place. Thus, as a measure of “the insect disturbance”, we will use a relative difference between the integral spectra (shown in Fig. 2c) in regions (3) and (1). This would mean a post-flashing reaction versus the initial state.

**Figure 3:**
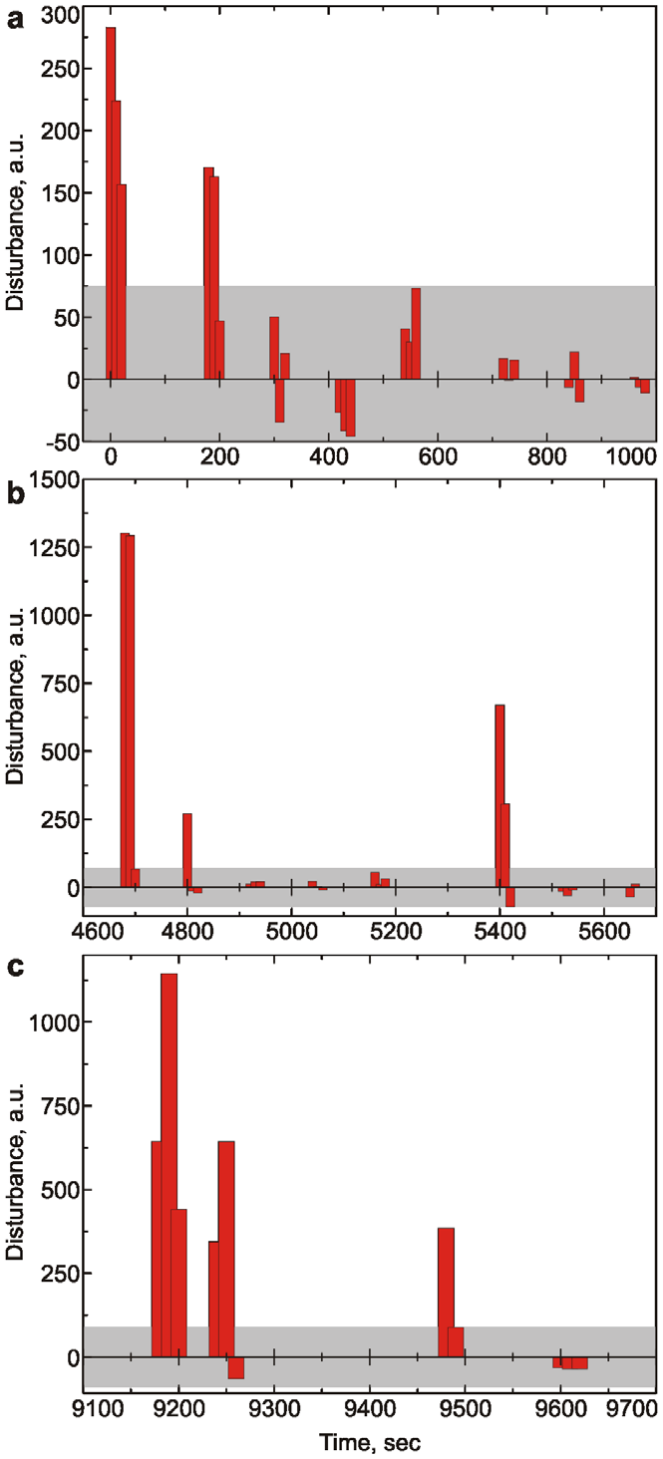
“Measure of disturbance” of the series of flashing (0.5–1 Hz) UV light of 375nm light. The gray region is the area of fluctuations of a presumably relaxed insect. The series of Fig. 2 are repeated several times with 2–3, 4–5 min and 1 hour beaks in-between.

We can now study how the beetle adopts to the light if we keep repeating the flashing sequence shown in Fig. 2. Each series to be repeated has 100 sec recorded data starting from rest (0–10sec), flashing light on (10–40sec), flashing light off; insect relaxation (40–70sec), and arriving to the state similar to the initial rest stage (70–100sec). The integral spectra– the sum of the Fourier amplitudes within the range of 40–100Hz was split into four regions as shown in Fig. 2c (I1, I2, I3, I4). As a measure of “the insect disturbance”, we used three relative differences (I3-I1)/I1*100%, where I3 was averaged with 40–50sec, 50–60 sec, and 60–70 sec (I1 was averaged 0–5 sec in all measurements). The series described above was repeated several times with 2–3, 4–5 min, and 1 hour beaks in-between the calculated insect disturbance is shown in Fig. 3. The gray region shown is the area of fluctuations of a presumably relaxed insect. This can be concluded based on the negative values adjacent to the values of the gray area. The negative values mean that the insect gets “quieter” than it was before turning the flashing light on. One can see that the beetles adapts to the disturbance of flashing light within 1–2 minutes (at the end of the second series of Fig. 3). 2–3 min breaks are not enough for the beetles to relax the disturbing action of the flashes. However, 4–5 minutes breaks lead the beetle to react to the flashing noticeably again. The beetle relaxes to the initial “alert” state after 1 hour breaks.

The observed process of adaptation and relaxation is presumably a combination of what might-be-called “learning”/“forgetting” and stress/relax. Because the insect spectrum recovers its character at the end of each 100 second measurement, possible contribution of stress is reversible (the possible stress is not severe). Interestingly, the time of adaptation and relaxation and back was slightly but consistently different for different beetles. It means that this type of experiment could be used to quantify the functions of nervous systems of individual insects.

### Beetle's sensitivity to light of different wavelengths

**Figure 4:**
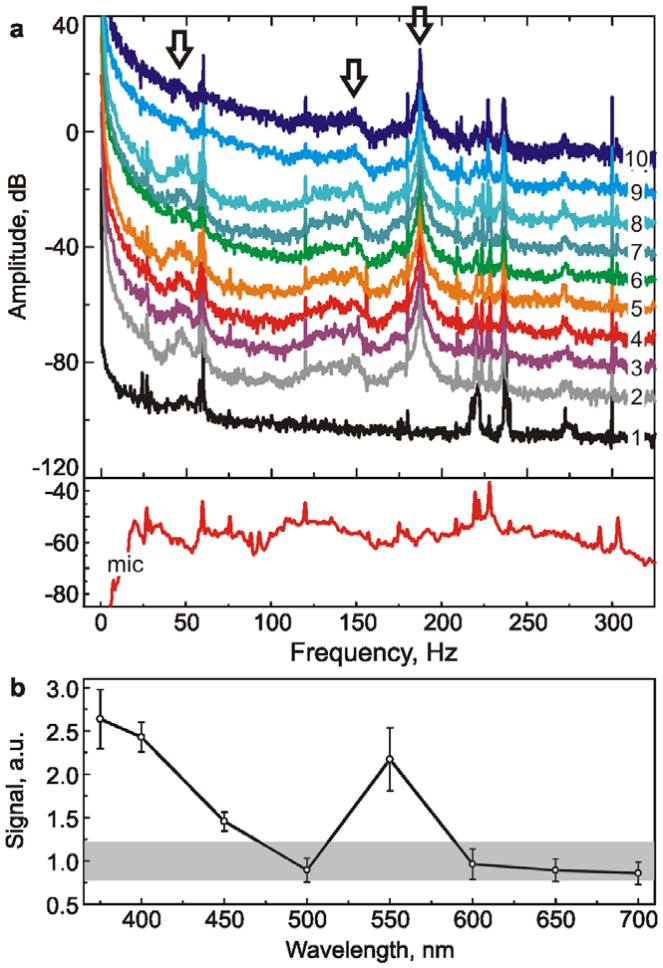
The beetle's spectral response to flashing light of different wavelengths. (a) Representative spectra measured for a beetle exposed to flashing monochromatic light of various wavelengths ranging from 700 nm (curve 3) to 375 nm (curve 10) with 50-nm step. Curves (1) and (2) are the reference spectra recorded on a dead and living (with no illumination) beetle, respectively. The vertical shift of 10 dB was added to each consequent spectrum except (1) for better visibility. The spectrum of the room noise collected from a microphone is shown in the bottom. Peaks typical for the beetle are (47, ∼150, and 187 Hz) shown with arrows. (b) Spectral amplitudes averaged in 10Hz windows within 10–100Hz range. The average value and one standard deviation are shown for each frequency. Grey area is the variation of the spectra for the beetle with no flashing light.

To study the beetle's sensitivity to light of different colors or wavelengths, we used a scheme similar to the previous experiment. To avoid the adaptation effect describe above, we started from near infrared light and finished with ultraviolet, from 700 nm to 375nm (As one will see, near infrared and red light do not disturb the insect). Fig. 4a shows representative spectra collected on a ladybird beetle exposed to the flashing monochromatic light of various wavelengths, from 700 nm (curve 3) to 375 nm (curve 10) with 50-nm step. Curves (1) and (2) are the reference spectra recorded on a dead and living (with no illumination) beetle, respectively. It is interesting to note that one can identify at least three frequencies typical for viable ladybird beetles here: 47, ∼150, 187 Hz (shown with the arrows in Fig. 4a) by comparing the recorded peaks with the noise signals. Correlating various features of the spectra and the wavelength of the flashing light, one can conclude about sensitivity of the ladybird beetle to the light of particular wavelength. The most robust feature of the spectra which correlated with the presence of flashing light was the spectral values in the range of 10–100Hz.

The dependence of the averaged value of the spectral amplitude in that range on the wavelength of flashing light, Fig. 4b, shows an interesting sensitivity of the beetle to light. Compared to the variation of the spectra for the beetle with no flashing light (the grey zone of Fig. 4b), one can see that the beetle has a high sensitivity in the range of near UV (375–400 nm) and around green (550 nm) light. It is known that beetles can distinguish illuminated and shaded areas, and seems specialized to make use of sky polarization in the UV region of the spectrum and/or the position of the sun as a course-stabilizing function during flights [21]. High sensitivity of bees to ultraviolet light is also known [20]. Thus, the reaction of ladybird beetles to the flashes of UV light can be expected from the previous results for beetles and bees. However, the blindness to the light around 500nm (emerald color) and high sensitivity to 550nm (green) color has not been reported before.

To conclude, the described approach is capable of detecting internal beats of insect organisms at subnanometer spatial and submilisecond spectral resolution by using an AFM probe, which can be located in different parts of the insects with a nanometer precision. Studying the spectra of recorded oscillations, one can learn the response of the insect organism to external stimuli in a non-invasive manner. This may shed light on unsolved problems of insect functions and behavior. The approach may lead to the emergence of what could be called “nanophysiology” of insects.

## Materials and Methods

### Atomic Force Microscopy (AFM) and Data Acquisition System

All vibrations of the insect surfaces were recorded by means of a Veeco Dimension 3100 AFM equipped with NanoScope V controller (all other versions of the controller could also be used). Veeco Silicone Nitride integrate DNP tips with spring constant k∼0.06 N/m were used in this study. The vertical position of the AFM cantilever was recorded. This signal was directly sampled using a National Instruments ADC 24-bit card (NI PCI-5922) card at 50 kHz. Simultaneously, the external sound was recorded by means of a wideband microphone. The sound was sampled using the second input channel of the same ADC card. LabView (by National Instruments) version 8.2 was used as the data collection interface.

### AFM Stage and Data Collection Method

**Figure 5:**
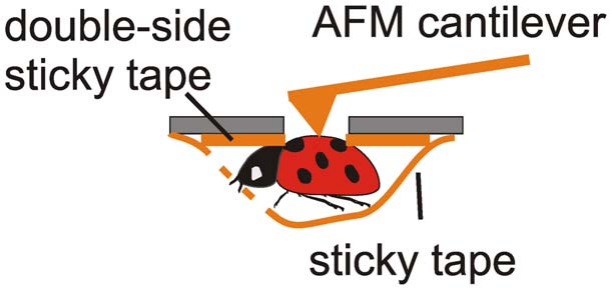
A special insect holder that restricts the motion of the insect.

The key element of the stage is a thin metallic membrane with an opening of a few millimeters in diameter. Specific diameter depends on the type of the insect of interest (in this work we used 5mm opening). The insect was attached from underneath the membrane, Fig. 1a, with the help of a Scotch tape. This minimizes the insect motion in vertical direction and virtually excludes any movement in lateral directions. An important part of using the Scotch tape was the double sticky tape surrounding the aperture of the membrane (shown in Fig. 5). This restricted lateral motions of the insect while keeping it relatively free with the Scotch tape (which might otherwise be damaging for the insect). The other important part is the sticky tape applied on the top of the membrane to restrict the vertical motion further. This is particular important when one deals with a sufficiently soft part of the insect.

An AFM tip was positioned on the top of the insect through the aperture in the holder membrane. The scan size was set to 0 nm and scan rate was set to 0.1Hz. To prevent a possible influence of the feedback, the scan feedback gain parameters were set between 0 to 0.01 for the integral, and to 0 for the proportional gain. The spectral signals shown in the paper were collected and both integral and proportional gains equal to 0.

### Other instrumentation

In the behavioral experiments, ladybird beetles were exposed to a flashing (frequency of 0.5–1 Hz) monochromatic light of different wavelengths, from deep red/near IR (700 nm) to the UV region (375 nm). A X-Cite 120 Fluorescence Illumination System and Jobin YVON 6/168V monochromator were used as a source of monochromatic light. A broad band RadioShack Super-Cardioid Dynamic Microphone 33-3042 was used. It has a frequency response of 50∼15000Hz at −72±3dB sensitivity.”

### Insects

*Hippodamia convergens* ladybird beetles (Hirt's Gardens, Granger, OH) were used in this work.

### The choice of the spectral region for analysis

**Figure 6:**
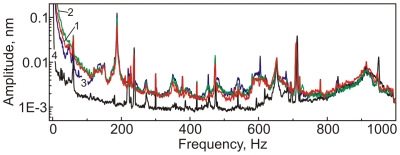
Representative spectra of vibrations of elytra of a ladybird beetle exposed to a flashing monochromatic light of wavelengths (1) 375 nm, (2) 550 nm, and (3) 700 nm. Black curve (4) shows the spectrum of a dead beetle. The shown spectra are averaged over a 100 sec interval.

Fig. 6 shows an extended region of spectral analysis of the recorded signal from ladybird beetles. The signals were collected from the surface of the beetle illuminated with flashing light of three different wavelengths. A spectrum collected from the same but dead beetle (recorded after several days) is shown as a reference. Substantial differences compared to the reference spectrum can be observed for the frequencies less than ∼500–600 Hz. One can see substantial artifacts at 650, 720, and 800–1000Hz. Comparing these frequencies with the sound spectrum recorded by microphone (not shown), we can conclude that all named artifacts are due to an intrinsic noise in the AFM instrument. Comparing the differences in the spectra corresponding to the three different wavelengths, one can see a substantial difference for the frequencies less than ∼100Hz.
